# Supplementary material for: The effects of genital myiasis on the diversity of the vaginal microbiota in female Bactrian camels
Source: BMC Vet Res. 2022 Mar 5;18:87. doi: 10.1186/s12917-022-03189-5 (PMC8897907; doi:10.1186/s12917-022-03189-5)
Supplement: Supplementary file 5 — Additional file 5. [file 12917_2022_3189_MOESM5_ESM.zip › MPL201709200_16s_yy/Treat1/css/js_css/pdfmake-master/examples/pdfs/columns_simple.pdf]

By default paragraphs are stacked one on top of (or actually - below) another.
It's possible however to split any paragraph (or even the whole document) into columns.

Here we go with 2 star-sized columns, with justified text and gap set to 20:

Lorem ipsum dolor sit amet, consectetur adipisicing elit. Malit profecta versatur nomine ocurreret multavit, officiis viveremus aeternum superstitio suspicor alia nostram, quando nostros congressus susceperant concederetur leguntur iam, vigiliae democritea tantopere causae, atilii plerumque ipsas potitur pertineant multis rem quaeri pro, legendum didicisse credere ex maluisset per videtis. Cur discordans praetereat aliae ruinae dirigentur orestem eodem, praetermittenda divinum. Collegisti, deteriora malint loquuntur officii cotidie finitas referri doleamus ambigua acute. Adhaesiones ratione beate arbitraretur detractis perdiscere, constituant hostis polyaeno. Diu concederetur.

Lorem ipsum dolor sit amet, consectetur adipisicing elit. Malit profecta versatur nomine ocurreret multavit, officiis viveremus aeternum superstitio suspicor alia nostram, quando nostros congressus susceperant concederetur leguntur iam, vigiliae democritea tantopere causae, atilii plerumque ipsas potitur pertineant multis rem quaeri pro, legendum didicisse credere ex maluisset per videtis. Cur discordans praetereat aliae ruinae dirigentur orestem eodem, praetermittenda divinum. Collegisti, deteriora malint loquuntur officii cotidie finitas referri doleamus ambigua acute. Adhaesiones ratione beate arbitraretur detractis perdiscere, constituant hostis polyaeno. Diu concederetur.

Star-sized columns have always equal widths, so if we define 3 of those, it'll look like this (make sure to scroll to the next page, as we have a couple of more examples):

Lorem ipsum dolor sit amet, consectetur adipisicing elit. Malit profecta versatur nomine ocurreret multavit, officiis viveremus aeternum superstitio suspicor alia nostram, quando nostros congressus susceperant concederetur leguntur iam, vigiliae democritea tantopere causae, atilii plerumque ipsas potitur pertineant multis rem quaeri pro, legendum didicisse credere ex maluisset per videtis. Cur discordans praetereat aliae ruinae dirigentur orestem eodem, praetermittenda divinum. Collegisti, deteriora malint loquuntur officii cotidie finitas referri doleamus ambigua acute. Adhaesiones ratione beate

Lorem ipsum dolor sit amet, consectetur adipisicing elit. Malit profecta versatur nomine ocurreret multavit, officiis viveremus aeternum superstitio suspicor alia nostram, quando nostros congressus susceperant concederetur leguntur iam, vigiliae democritea tantopere causae, atilii plerumque ipsas potitur pertineant multis rem quaeri pro, legendum didicisse credere ex maluisset per videtis. Cur discordans praetereat aliae ruinae dirigentur orestem eodem, praetermittenda divinum. Collegisti, deteriora malint loquuntur officii cotidie finitas referri doleamus ambigua acute. Adhaesiones ratione beate

Lorem ipsum dolor sit amet, consectetur adipisicing elit. Malit profecta versatur nomine ocurreret multavit, officiis viveremus aeternum superstitio suspicor alia nostram, quando nostros congressus susceperant concederetur leguntur iam, vigiliae democritea tantopere causae, atilii plerumque ipsas potitur pertineant multis rem quaeri pro, legendum didicisse credere ex maluisset per videtis. Cur discordans praetereat aliae ruinae dirigentur orestem eodem, praetermittenda divinum. Collegisti, deteriora malint loquuntur officii cotidie finitas referri doleamus ambigua acute. Adhaesiones ratione beate

arbitraretur detractis perdiscere, constituant hostis polyaeno. Diu concederetur.

arbitraretur detractis perdiscere, constituant hostis polyaeno. Diu concederetur.

arbitraretur detractis perdiscere, constituant hostis polyaeno. Diu concederetur.

You can also specify accurate widths for some (or all columns). Let's make the first column and the last one narrow and let the layout engine divide remaining space equally between other star-columns:

Lorem ipsum dolor sit amet, consectetur adipisicing elit. Malit profecta versatur nomine ocurreret multavit, officiis viveremus aeternum superstitio suspicor alia nostram, quando nostros congressus susceperant concederetur leguntur iam, vigiliae democritea tantopere causae, atilii plerumque ipsas potitur pertineant multis rem quaeri pro, legendum didicisse credere ex maluisset per videtis. Cur discordans praetereat aliae ruinae dirigentur orestem eodem, praetermittenda divinum. Collegisti,

Lorem ipsum dolor sit amet, consectetur adipisicing elit. Malit profecta versatur nomine ocurreret multavit, officiis viveremus aeternum superstitio suspicor alia nostram, quando nostros congressus susceperant concederetur leguntur iam, vigiliae democritea tantopere causae, atilii plerumque ipsas potitur pertineant multis rem quaeri pro, legendum didicisse credere ex maluisset per videtis. Cur discordans praetereat aliae ruinae dirigentur orestem eodem, praetermittenda divinum. Collegisti, deteriora malint loquuntur officii cotidie finitas referri doleamus ambigua acute. Adhaesiones ratione beate arbitraretur detractis perdiscere, constituant hostis polyaeno. Diu concederetur.

Lorem ipsum dolor sit amet, consectetur adipisicing elit. Malit profecta versatur nomine ocurreret multavit, officiis viveremus aeternum superstitio suspicor alia nostram, quando nostros congressus susceperant concederetur leguntur iam, vigiliae democritea tantopere causae, atilii plerumque ipsas potitur pertineant multis rem quaeri pro, legendum didicisse credere ex maluisset per videtis. Cur discordans praetereat aliae ruinae dirigentur orestem eodem, praetermittenda divinum. Collegisti, deteriora malint loquuntur officii cotidie finitas referri doleamus ambigua acute. Adhaesiones ratione beate arbitraretur detractis perdiscere, constituant hostis polyaeno. Diu concederetur.

Lorem ipsum dolor sit amet, consectetur adipisicing elit. Malit profecta versatur nomine ocurreret multavit, officiis viveremus aeternum superstitio suspicor alia nostram, quando nostros congressus susceperant concederetur leguntur iam, vigiliae democritea tantopere causae, atilii plerumque ipsas potitur pertineant multis rem quaeri pro, legendum didicisse credere ex maluisset per videtis. Cur discordans praetereat aliae ruinae dirigentur orestem eodem, praetermittenda divinum. Collegisti,

deteriora malint loquuntur officii cotidie finitas referri doleamus ambigua acute. Adhaesiones ratione beate arbitraretur detractis perdiscere, constituant hostis polyaeno. Diu concederetur.

deteriora malint loquuntur officii cotidie finitas referri doleamus ambigua acute. Adhaesiones ratione beate arbitraretur detractis perdiscere, constituant hostis polyaeno. Diu concederetur.

We also support auto columns. They set their widths based on the content:

auto column

This is a star-sized column. It should get the remaining space divided by the number of all star-sized columns.

this one has specific width set to 50

another auto column

This is a star-sized column. It should get the remaining space divided by the number of all star-sized columns.

If all auto columns fit within available width, the table does not occupy whole space:

val1	val2	value3	value 4

Another cool feature of pdfmake is the ability to have nested elements. Each column is actually quite similar to the whole document, so we can have inner paragraphs and further divisions, like in the following example:

Lorem ipsum dolor sit amet, consectetur adipisicing elit. Conveniunt quieti extremum severitatem disseretur virtute locum virtus declarant. Greges telos detrimenti persius possint eripuit appellat democrito suscipere existimant. Facere usus levitatibus confirmavit, provincia rutilius libris accommodare valetudinis ignota fugienda arbitramur falsarum commodius. Voluptas summis arbitrarer cognitio temperantiamque, fuit posidonium pro assueverit animos

As you can see in the document definition - this column is not defined with an object, but an array, which means it's treated as an array of paragraphs rendered one below another.
Just like on the top-level of the document. Let's try to divide the remaing space into 3 star-sized columns:

Lorem ipsum dolor sit amet, consectetur adipisicing elit. Malit profecta versatur nomine ocurreret multavit, officiis viveremus aeternum superstitio suspicor alia nostram, quando nostros congressus susceperant

Lorem ipsum dolor sit amet, consectetur adipisicing elit. Malit profecta versatur nomine ocurreret multavit, officiis viveremus aeternum superstitio suspicor alia nostram, quando nostros congressus susceperant

Lorem ipsum dolor sit amet, consectetur adipisicing elit. Malit profecta versatur nomine ocurreret multavit, officiis viveremus aeternum superstitio suspicor alia nostram, quando nostros congressus susceperant

inferiorem, affecti honestum ferreum cum tot nemo ius partes dissensio opinor, tuum intellegunt numeris ignorant, odia diligenter licet, sublatum repellere, maior ficta severa quantum mortem. Aut evertitur impediri vivamus.

concederetur leguntur iam, vigiliae democritea tantopere causae, atilii plerumque ipsas potitur pertineant multis rem quaeri pro, legendum didicisse credere ex maluisset per videtis. Cur discordans praetereat aliae ruinae dirigentur orestem eodem, praetermittenda divinum. Collegisti, deteriora malint loquuntur officii cotidie finitas referri doleamus ambigua acute. Adhaesiones ratione beate arbitraretur detractis perdiscere, constituant hostis polyaeno. Diu concederetur.

concederetur leguntur iam, vigiliae democritea tantopere causae, atilii plerumque ipsas potitur pertineant multis rem quaeri pro, legendum didicisse credere ex maluisset per videtis. Cur discordans praetereat aliae ruinae dirigentur orestem eodem, praetermittenda divinum. Collegisti, deteriora malint loquuntur officii cotidie finitas referri doleamus ambigua acute. Adhaesiones ratione beate arbitraretur detractis perdiscere, constituant hostis polyaeno. Diu concederetur.

concederetur leguntur iam, vigiliae democritea tantopere causae, atilii plerumque ipsas potitur pertineant multis rem quaeri pro, legendum didicisse credere ex maluisset per videtis. Cur discordans praetereat aliae ruinae dirigentur orestem eodem, praetermittenda divinum. Collegisti, deteriora malint loquuntur officii cotidie finitas referri doleamus ambigua acute. Adhaesiones ratione beate arbitraretur detractis perdiscere, constituant hostis polyaeno. Diu concederetur.

Oh, don't forget, we can use everything from styling examples (named styles, custom overrides) here as well.

For instance - our next paragraph will use the 'bigger' style (with fontSize set to 15 and italics - true). We'll split it into three columns and make sure they inherit the style:

First column (BTW - it's defined as a single string value. pdfmake will turn it into appropriate structure automatically and make sure it inherits the styles

In this column, we've overriden fontSize to 20. It means the content should have italics=true (inherited from the

Last column does not override any styling properties, but applies a new style (header) to itself. Eventually - texts here have italics=true (from

style) and be a little bit bigger

bigger) and derive fontSize from the style. OK, but which one? Both styles define it. As we already know from our styling examples, multiple styles can be applied to the element and their order is important. Because 'header' style has been set after 'bigger' its fontSize takes precedence over the fontSize from 'bigger'. This is how it works. You will find more examples in the unit tests.

Wow, you've read the whole document! Congratulations :D
